# Hematopoietic lineage cell-specific protein 1 (HS1), a hidden player in migration, invasion, and tumor formation, is over-expressed in ovarian carcinoma cells

**DOI:** 10.18632/oncotarget.25975

**Published:** 2018-08-24

**Authors:** Yoshihiro Koya, Wenting Liu, Yoshihiko Yamakita, Takeshi Senga, Kiyosumi Shibata, Mamoru Yamashita, Akihiro Nawa, Fumitaka Kikkawa, Hiroaki Kajiyama

**Affiliations:** ^1^ Bell Research Center, Department of Obstetrics and Gynecology Collaborative Research, Nagoya University Graduate School of Medicine, Showa-ku, Nagoya, Japan; ^2^ Bell Research Center for Reproductive Health and Cancer, Nagoya, Aichi, Japan; ^3^ Yahagigawa Hospital, Anjo, Aichi, Japan; ^4^ Department of Obstetrics and Gynecology, Fujita Health University, Banbuntane Hotokukai Hospital, Nakagawa-ku, Nagoya, Japan; ^5^ Department of Obstetrics and Gynecology, Nagoya University Graduate School of Medicine, Showa-ku, Nagoya, Japan

**Keywords:** epithelial ovarian cancer (EOC), migration/invasion, cytoskeletal protein, tumor formation, prognostic factor

## Abstract

Hematopoietic lineage cell-specific protein 1 (HS1), which is the hematopoietic homolog of cortactin, is an actin-binding protein and Lyn substrate. It is upregulated in several cancers and its expression level is associated with increased cell migration, metastasis, and poor prognosis. Here we investigated the expression and roles of HS1 in ovarian carcinoma cells. We analyzed the expression of HS1 in 171 ovarian cancer specimens and determined the association between HS1 expression and clinicopathological characteristics, including patient outcomes. In patients with stage II–IV disease, positive HS1 expression was associated with significantly worse overall survival than negative expression (*P* < 0.05). HS1 was localized in invadopodia in some ovarian cancer cells and was required for invadopodia formation. Migration and invasion of ovarian cancer cells were suppressed by down-regulation of HS1, but increased in cells that over-expressed exogenous HS1. Furthermore, ovarian cancer cells that expressed HS1 shRNA exhibited reduced tumor formation in a mouse xenograft model. Finally, we found that tyrosine phosphorylation of HS1 was essential for cell migration and invasion. These findings show that HS1 is a useful biomarker for the prognosis of patients with ovarian carcinoma and is a critical regulator of cytoskeleton remodeling involved in cell migration and invasion.

## INTRODUCTION

Tumor metastasis involves multiple steps, as follows: 1) carcinoma development and acquisition of invasive potential; 2) detachment of metastatic carcinoma cells from the primary tumor, followed by migration and invasion of the basement membrane; 3) intravasation of metastatic carcinoma cells; 4) transport of metastatic carcinoma cells through lymphatic or blood vessels; 5) extravasation of metastatic carcinoma cells at secondary sites; and 6) invasion of secondary tissues and formation of micro- or macro-metastasis [1, 2]. Of these steps, invasion plays a critical role since it is a prerequisite for tumor metastasis. In other words, carcinoma cells that do not have the ability to invade grow only in the primary site. Accentuation of cell motility, which is associated with the acquisition of invasive potential in carcinoma cells, is induced by a variety of mechanisms [3–5]. Carcinoma cells must destroy the basement membrane surrounding the tumor itself in order for them to infiltrate into the tumor stroma. Invadopodia degrade the extracellular matrix of the basement membrane to promote tumor cell invasion.

CTTN is a cytoskeletal protein whose over-expression increases tumor aggressiveness by promoting tumor migration, invasion, and metastasis. It is amplified in many types of solid tumors, including melanoma, ovarian, breast, and colorectal cancers [6–8]. Many studies have analyzed the contribution of CTTN to promoting cell motility and invasion [8–10]. During the invasion process, CTTN plays an essential role in invadopodia, actin-rich subcellular protrusions associated with degradation of the extracellular matrix by cancer cells [11, 12]. CTTN binds to the Arp2/3 complex and promotes actin assembly by helping to form and stabilize actin filament branches. It has also been reported that CTTN is associated with tumor progression and poor prognosis in many malignancies [13–15].

A homologue of CTTN is hematopoietic lineage cell-specific protein 1 (HS1, also called LckBP1 [16]). The *HS1* gene was originally reported to be expressed exclusively in hematopoietic lineage cells [17], while CTTN is found in all cell types but these [18]. Although a subsequent study reported that HS1 is not restricted to cells of hematopoietic origin [19], it is still unknown what role HS1 plays in non-hematopoietic cells. HS1 has numerous functions in hematopoietic cells; in particular, it contributes to B- and T-cell antigen receptor-mediated signal transduction [20, 21], and promotes both Arp 2/3 complex-mediated actin polymerization [22] and the migration of natural killer cells [23]. In addition, HS1 regulates trafficking and homing in chronic lymphocytic leukemia, and contributes to tissue invasion and infiltration [24]. It has also been reported that HS1 is abnormally expressed in B-cell chronic lymphocytic leukemia and correlates with poor survival of patients [25, 26]. Given the above, the question arises whether HS1 contributes to cell migration and invasion and correlates with prognosis in solid tumors.

Among gynecological malignancies, epithelial ovarian carcinoma (EOC) is the leading cause of death worldwide [27]. Recently, the numbers of EOC patients and deaths from EOC have been increasing in Japan [28]. Ovarian cancer cells (OCCs) often metastasize not by lymphogenous or hematogenous routes, but rather via ascites formation throughout the peritoneal cavity, including the omentum and parenchyma of various organs [29]. Though there are many reviews regarding the mechanisms of cancer cell metastasis [2, 30, 31], the specific details involved remain unknown.

## RESULTS

### The level of HS1 is correlated with prognosis of ovarian cancer patients

**Figure 1:**
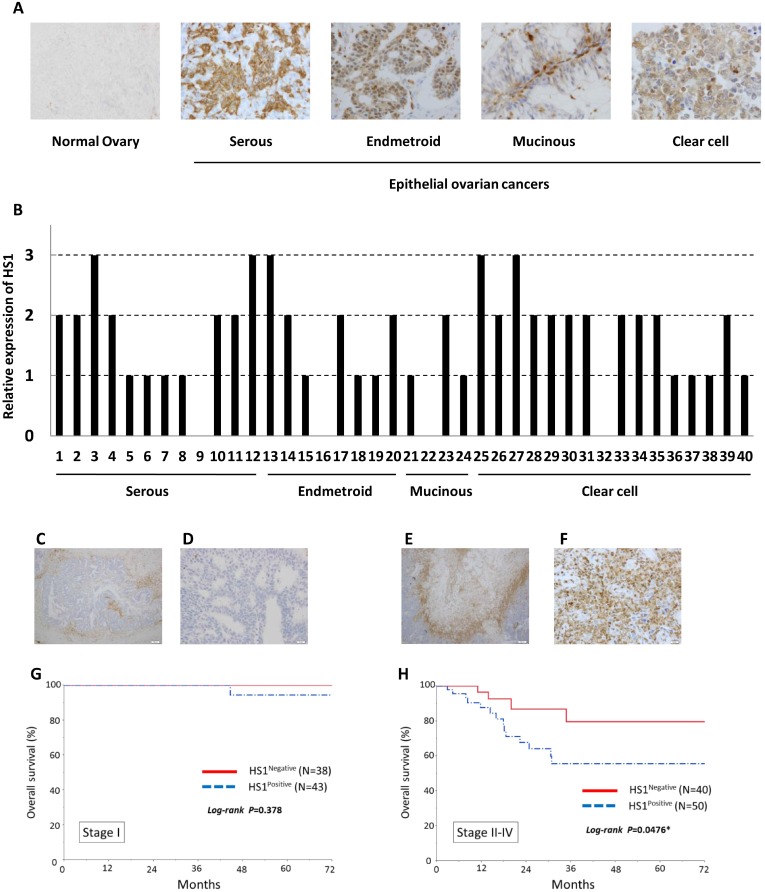
HS1 is expressed in ovarian cancer tissues and its expression is relevant to overall survival (**A**) Analysis of HS1 expression was performed in normal ovarian tissue and EOC tissues. (**B**) Expression of HS1 is shown as a bar graph. The data were extracted at random from 171 ovarian cancer specimens. (**C**–**F**) Immunohistochemical analysis of HS1 in ovarian cancer tissues; representative micrographs of (C and D) HS1-negative lesions and (E and F) HS1-positive lesions are shown. (**G** and **H**) Overall survival rates of ovarian cancer patients with tumors exhibiting HS1 expression. Kalan–Meier survival curves are shown according to the immunoexpression of HS1 and stratified by International Federation of Gynecology and Obstetrics (FIGO) ovarian cancer stage: (G) stage I and (H) stages II–IV. Stage II–IV patients exhibiting HS1 expression had significantly poorer carcinoma-specific survival (*P* = 0.0476).

We first performed immunohistochemical detection of HS1 in 171 ovarian cancer specimens. While HS1 was not expressed in normal ovarian tissue, it was highly expressed in various types of epithelial ovarian cancers (Figure 1A and 1B). HS1 was detected in the cell cytoplasm of OCCs (Figure 1C–1F). In several cases, HS1 expression in the tumor stroma was found to be higher than that in tumor cells. Next, we analyzed whether there was correlation between HS1 expression and ovarian cancer prognosis. In patients with stage I disease, no correlation was observed between the level of HS1 expression and OS (Figure 1G). In patients with stage II–IV disease, however, Kaplan–Meier analysis showed that positive HS1 expression was associated with a significantly shorter OS than negative HS1 expression (*P* < 0.05, Figure 1H). These data demonstrated that HS1 was over-expressed in ovarian cancer tissues and its expression was correlated closely with poor OS of patients with ovarian cancer.

### HS1 is expressed in highly invasive OCCs

**Figure 2:**
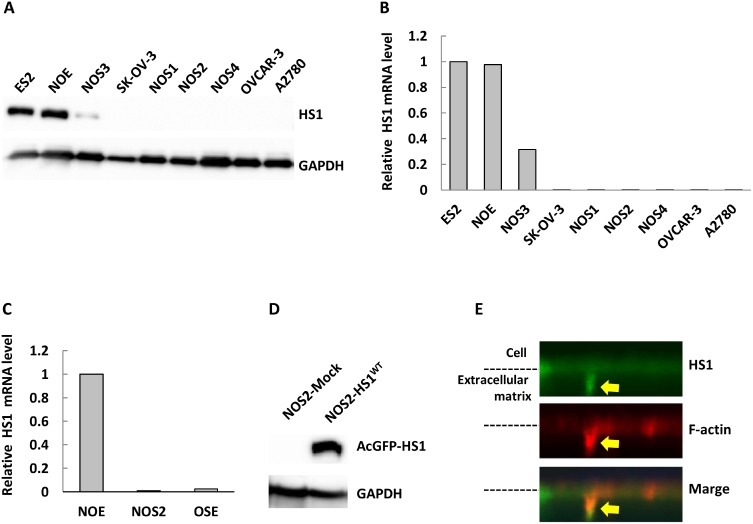
HS1 is expressed in highly invasive cells and is localized in invadopodia (**A**) Cells were lysed and immunoblot analysis was performed with an antibody against HS1. GAPDH was used as a loading control. (**B**) Total RNA was isolated from cells and used to synthesize cDNA. Quantitative PCR was used to measure the expression levels of the *HS1* gene. *GAPDH* was used for normalization. (**C**) Human ovarian surface epithelial cell (OSE) total RNA was purchased from Cosmo Bio. (**D**) Lysates extracted from NOS2-AcGFP-Mock and -AcGFP-HS1^WT^ were used to perform immunoblot analysis with an antibody against HS1. GAPDH was used as a loading control. (**E**) NOS2-AcGFP-HS1^WT^ cells were cultured overnight on gelatin-coated coverslips; the cells were then fixed and stained with phalloidin-594. A FluoView (Olympus) confocal microscope was used to acquire the photographs and reconstruct 3D images. The yellow arrow shows an invadopodium.

Next, we used quantitative real-time PCR (qPCR) and immunoblot analysis to analyze HS1 expression in a panel of OCC lines. As shown in Figure 2A and 2B, three of nine cell lines expressed HS1 protein and mRNA. One cell line, NOS3, exhibited low levels of both, while six cell lines showed no HS1 expression. Interestingly, ES2 and NOE, the two cells lines that most highly expressed HS1, are highly invasive *in vitro* and have high tumorigenicity *in vivo* [32, 33]. No HS1 expression was detected in human ovarian surface epithelial cells by qPCR (Figure 2C). These results suggest that expression of HS1 may correlate with invasiveness and tumorigenicity.

### HS1 is localized in invadopodia in OCCs

Next, we analyzed the localization of HS1 in OCC lines. HS1 was found in the cytoplasmic region of ES2 cells (Supplementary Figure 1). Since CTTN was found to localize in invadopodia [34], and HS1 is a homolog of CTTN in hematopoietic cells, we investigated whether HS1 localized in invadopodia in OCC lines. NOS2-AcGFP-HS1 cells were established (Figure 2D) in this proposed experiment, because ES2 and NOE cells were not suitable for this analysis. As shown in Figure 2E, HS1 co-localized with F-actin in an invadopodium (yellow arrow). This result suggests that HS1 localizes in invadopodia in OCCs.

### HS1 is required for the promotion of migration and invasion of OCCs

**Figure 3:**
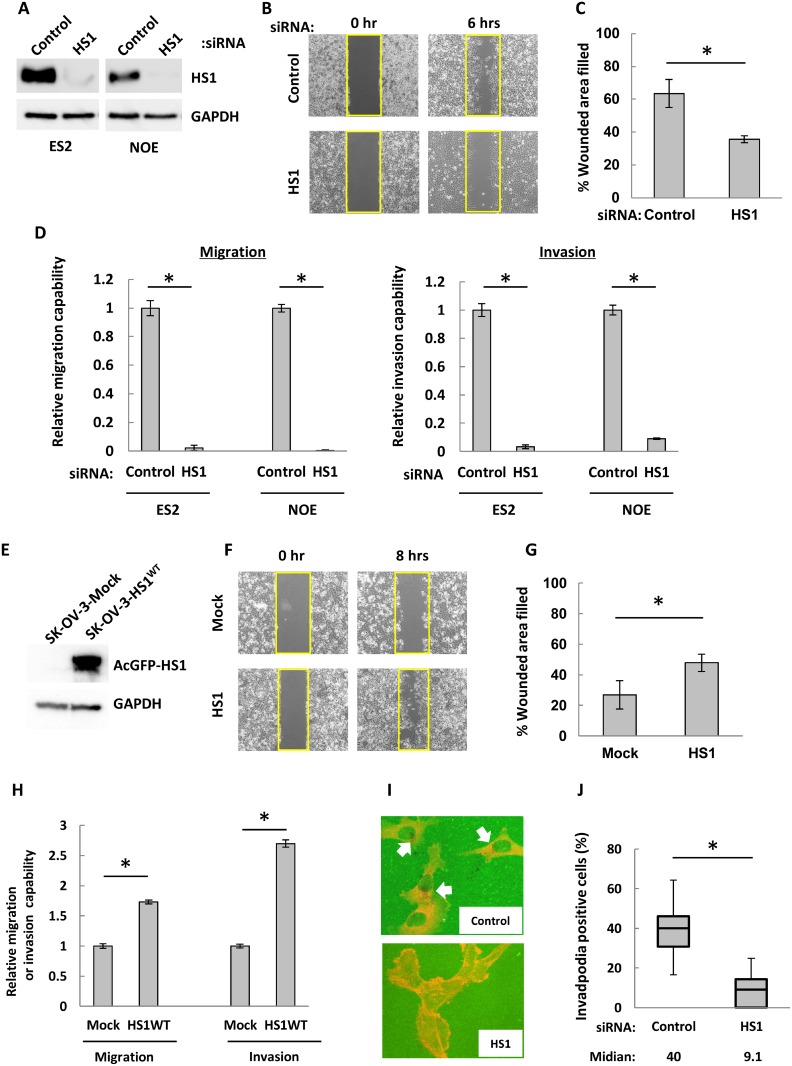
In ovarian cancer cells, HS1 contributes to wound-healing, migration, and invasion abilities as well as to invadopodia formation (**A**) After transfection with HS1 siRNA (HS1-268 and -965, 10 nM each) or control (20 nM) [64] cell lysates were used in immunoblot analysis to confirm knock-down of HS1. (**B**) ES2 cells were transfected with HS1 siRNA or control for 48 hours. Cells were then incubated overnight in a Culture-Insert 2 Well, and at 0 and 6 hours after removal from the well, photographs were acquired with an inverted microscope (Olympus). (**C**) The percentages of original wound areas that had healed were measured by ImageJ software using acquired photographs, and are shown in the bar graph. (**D**) Cells were transfected with siRNA and then transwell migration and invasion assays were performed. After 22 hours, cells were fixed and stained and then photographs were acquired. The migrating and invading cells were counted using ImageJ software, and the relative migration or invasive abilities are shown (the abilities of cells transfected with control siRNA were used as a reference, with a value of 1). Each experiment was performed in triplicate. The bars indicate the mean ± s.d. ^*^P < 0.05 (*t*-test). (**E**) Lysates extracted from SK-OV-3-AcGFP-Mock and -AcGFP-HS1^WT^ were used to perform immunoblot analysis with an antibody against HS1. GAPDH was used as a loading control. (**F**) Cells were cultured overnight in a Culture-Insert 2 Well; at 0 and 8 hours after removal from the well, photographs were acquired with an inverted microscope (Olympus). (**G**) The percentages of original wound areas that had healed were measured by ImageJ software based on the acquired photographs, and are shown in the bar graph. (**H**) Transwell migration and invasion assays were performed. After 22 hours, cells were fixed and stained and then photographs were acquired. The migrating and invading cells were counted using ImageJ software, and the relative migration or invasive abilities are shown (the abilities of mock transfected cells were used as a reference, with a value of 1). Representative images of migration and invasion assays are shown. Each experiment was performed in triplicate. The bars indicate the mean ± s.d. ^*^P < 0.05 (*t*-test). (**I**) NOE cells were transfected with HS1 siRNA (HS1-268 and -965, 10 nM each) or control (20 nM) for 48 hours; cells were then seeded overnight on FITC-conjugated gelatin (green) with a 5-nM MMP inhibitor. After the inhibitor was removed, cells were cultured for 6 hours and were then fixed and stained with phalloidin (red). The black areas underneath the cells are gelatin degradation spots. Invadopodia were defined by the co-localization of a gelatin degradation spot (black spot) with F-actin (red spot), and are indicated by white arrows. (**J**) Photographs were acquired by a confocal microscope, and the total number of cells and the number of invadopodia-positive cells were counted. The percentages of invadopodia-positive cells are shown. The bars indicate the mean ± s.d. ^*^P < 0.05 (*t*-test).

CTTN promotes cell migration, invasion and tumor metastasis in many solid tumors [35, 36]. We therefore used siRNA techniques to investigate the impact of HS1 on the wound-healing, invasion, and migration abilities of OCCs. We designed siRNA against HS1, and these siRNA were transfected into cells from the OCC lines ES2 and NOE. Forty-eight hours after siRNA transfection, both cell lines demonstrated markedly decreased HS1 expression (Figure 3A) and showed morphological changes that were similar to those of NOS2 cells (Supplementary Figure 2). NOS2 cells did not exhibit invasive ability in a Matrigel chamber (data not shown). In ES2 cells with decreased HS1, a wound-healing assay showed that the percentage of the original wound area that had healed was around 35%, compared to 63% in cells transfected with control siRNA (Figure 3B and 3C). Following the decrease of HS1, migration and invasion abilities were decreased dramatically in both ES2 and NOE cells (Figure 3D). Finally, cells transfected with siRNA against HS1 showed no difference in CTTN expression compared to controls (Supplementary Figure 3). These results suggest that HS1 strongly contributes to the wound-healing, migration, and invasion abilities of OCCs.

To further confirm the critical role of HS1 in cell migration and invasion, we generated SK-OV-3 cells that constitutively expressed either AcGFP-HS1^WT^ or AcGFP. SK-OV-3 cells did not show detectable level of endogenous HS1 (Figure 3E). A wound healing assay revealed enhanced migration of AcGFP-HS1^WT^ cells compared to AcGFP cells (Figure 3F and 3G). A transwell assay also showed increased migration and invasion resulting from exogenous expression of AcGFP-HS1^WT^ (Figure 3H). These results show that HS1 plays an important role in migration and invasion of OCCs.

### HS1 maintains the levels of LEF1 and ZEB1 in OCCs

Morphological changes induced by HS1 knockdown made us speculate that HS1 is associated with epithelial-mesenchymal transition (EMT) (Supplementary Figure 4). During EMT, epithelial cells lose E-cadherin expression and cell-cell contact, acquire a spindle-like shape, and gain migration and invasion capabilities. HS1 and control siRNAs were transfected into ES2 and NOE cells, and RNAs were extracted to measure the expression of E-cadherin (epithelial cell marker) and N-cadherin (mesenchymal cell marker) mRNAs. Cells depleted of HS1 showed an increase of E-cadherin mRNA, whereas N-cadherin mRNA, was decreased by HS1 knockdown (Supplementary Figure 5). We next investigated whether there was any change in the expression of two EMT-associated transcription factors, LEF1 and ZEB1 [37–40]. We found that the expression of LEF1 and ZEB1 mRNAs was decreased by HS1 knockdown (Supplementary Figure 6). These results suggest that HS1 is required for LEF1 and ZEB1 expression to maintain the mesenchymal phenotype of OCCs.

### HS1 is required for invadopodia formation in OCCs

Next, we assessed the relation between HS1 expression and invadopodia formation. In a fluorescein isothiocyanate (FITC)-gelatin degradation assay, NOE cells transfected with siRNA against HS1 showed a significant reduction in the number of invadopodia-positive cells (median, 9.1) compared with cells transfected with control siRNA (median, 40) (Figure 3I and 3J). According to these results, HS1 plays an important role in invadopodia formation in OCCs.

### HS1 promotes tumorigenicity *in vivo*

**Figure 4:**
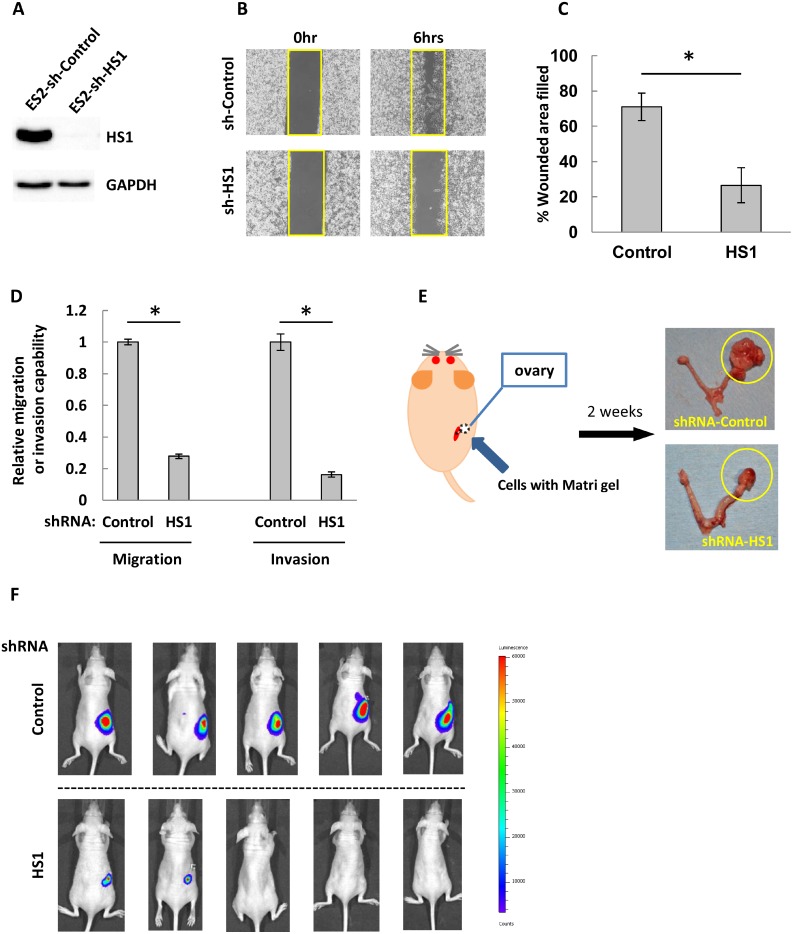
HS1 plays an important role in tumorigenesis in ovarian cancer cells (**A**) Lysates extracted from ES2-shRNA-Control and -shRNA-HS1 were used to perform immunoblot analysis with an antibody against HS1. GAPDH was used as a loading control. (**B**) Cells were cultured overnight in a Culture-Insert 2 Well; at 0 and 6 hours after removal from the well, photographs were acquired by microscopy. (**C**) The percentages of original wound areas that had healed were measured by ImageJ software based on the acquired photographs, and are shown in the bar graph. (**D**) Cells were transduced with shRNA and then transwell migration and invasion assays were performed. After 22 hours, cells were fixed and stained and then photographs were acquired. The migrating and invading cells were counted using ImageJ software, and the relative migration or invasive abilities are shown (the abilities of cells transfected with control shRNA were used as a reference, with a value of 1). Each experiment was performed in triplicate. The bars indicate the mean ± s.d. ^*^P < 0.05 (*t*-test). (**E**) The orthotopic inoculation scheme. Cells and Matrigel were injected into murine ovaries through a retroperitoneal approach from the dorsal flank. After inoculation for 2 weeks, injected cells demonstrated growth in murine ovaries, as indicated by the yellow circles. (**F**) After 2 weeks, tumor growth in inoculated mice was analyzed. Mice were intraperitoneally injected with luciferin then assessed with an IVIS imaging system. The upper row shows mice inoculated with ES2-shControl and the lower row shows mice inoculated with ES2-shHS1.

We analyzed whether HS1 contributes to tumorigenic potential *in vivo*. First, we established cells with constitutively decreased HS1. ES2 cells were transduced with shRNA against an HS1-expressing retroviral vector or an empty retroviral vector as a control. The cells transduced with HS1 shRNA showed a marked decrease of HS1 expression (Figure 4A), and the percentage of the original wound area that had healed was around 26.5%, which was less than half the percentage in controls (Figure 4B and 4C). The cells with decreased HS1 also demonstrated significantly reduced migration and invasion abilities compared with controls (Figure 4D). Next, we analyzed the *in vivo* tumorigenicity of these transduced cells. Two inoculation methods were used: the first was intraperitoneal inoculation as a peritoneal dissemination model of ovarian carcinoma, and the second was injection of ovaries (orthotopic inoculation) as a model of primary ovarian cancer (Figure 4E). In the peritoneal dissemination model, inoculation with control shRNA cells resulted in pronounced intraperitoneal proliferation in mice, whereas inoculation with HS1 shRNA cells demonstrated limited tumor growth (Supplementary Figure 7). In the orthotopic inoculation model, ovaries injected with control shRNA were much larger than those injected with HS1 shRNA (Figure 4E and 4F). These results suggest that HS1 is highly tumorigenic *in vivo*.

### HS1 is more involved than CTTN in the migration and invasion of OCCs

The HS1 homolog CTTN is also expressed in ovarian cancer and is reported to contribute to cell migration. To determine the role of CTTN, we first examined its expression in multiple OCC lines by immunoblot analysis. As shown in Supplementary Figure 8, six of nine cell lines expressed detectable levels of the CTTN protein. ES2 and NOE showed high expression of CTTN, and we therefore compared CTTN with HS1 in terms of their roles in cell migration and invasion. Transfection of CTTN siRNA significantly suppressed CTTN expression in both cell lines. Migration and invasion of both cell lines were reduced by CTTN knockdown; however, the suppression was not as significant as that following HS1 depletion (Supplementary Figures 9 and 10). This lesser suppression of migration and invasion may result from the relatively lower amount of CTTN in ES2 and NOE cells; thus, we checked the levels of HS1 and CTTN mRNAs in ES2 and NOE cells using absolute quantification PCR (Supplementary Figure 11). The amount of CTTN mRNA was higher than that of HS1 mRNA in the four cell lines we examined. These results indicate that HS1 contributes more than CTTN to OCC migration and invasion.

### Phosphorylation of HS1 is a prerequisite for its functions in OCC invasion and migration

**Figure 5:**
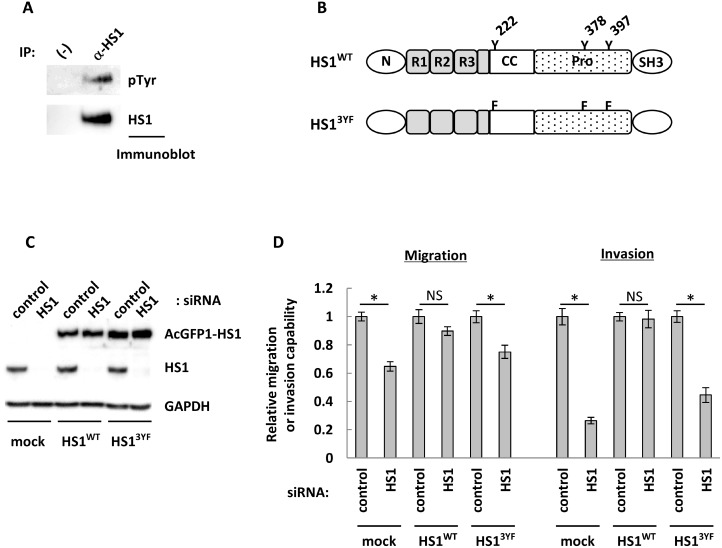
Phosphorylation of tyrosine residues may contribute to the impact of HS1 on cell migration and invasion abilities (**A**) A portion of ES2 cell lysates was set aside as input, shown as (−). The remainders of the lysates were subjected to precipitation with an anti-HS1 antibody (CST). The precipitates were immunoblotted with anti-HS1 (BD) or anti-pTyr (Millipore) antibodies. (**B**) The amino acid sequence of HS1 is divided into five domains, specifically N (NTA domain, interaction with Arp2/3), R1–R3 (interaction with F-actin), CC (coiled-coil region), Pro (proline-rich region), and SH3 (SH3 domain) [58]. The construction of HS1^WT^ and HS1^3YF^: HS1^WT^ has three tyrosine residues (Y) that are phosphorylation sites, and these residues were mutated to phenylalanine (F) in HS1^3YF^. (**C**) After transfection with siRNA HS1-1788 (20 nM) or control (20 nM), cell lysates were used for immunoblot analysis to confirm knock-down of endogenous HS1. GAPDH was used as a loading control. (**D**) Following transfection with siRNAs, transwell migration and invasion assays were performed. After 22 hours, cells were fixed and stained and then photographs were acquired. The migrating and invading cells were counted using ImageJ software, and the relative migration or invasive abilities are shown (cells transfected with control siRNA were used as a reference, with a value of 1). Each experiment was performed in triplicate. The bars indicate the mean ± s.d. ^*^P < 0.05, NS: not significant (*t*-test).

Previous reports have shown that tyrosine phosphorylation of HS1 promoted actin remodeling in leukocyte [21]. In addition, phosphorylation of tyrosine at positions 222, 378, and 397 was required for migration of natural killer cells [23]. We investigated whether tyrosine phosphorylation of HS1 played any role in promoting the migration and invasion of OCCs. To determine whether tyrosine phosphorylation of HS1 occurred in OCCs, HS1 was immunoprecipitated from ES2 cell lysate and subjected to immunoblot analysis with an anti-phosphotyrosine antibody (PY20). HS1 was clearly tyrosine-phosphorylated in ES2 cells (Figure 5A). Phosphorylation of the tyrosine residue at position 397 (Tyr397) in HS1 is essential for adhesion of natural killer cells to the integrin ligand ICAM-1 [41]. Treatment of ES2 and NOE2 cells with vanadate, a phosphatase inhibitor, induced phosphorylation of Tyr397, indicating tyrosine phosphorylation of endogenous HS1 in OCCs (Supplementary Figure 12). Lyn is considered to be the kinase of HS1, and we showed that it was expressed in all OCCs evaluated in this study (Supplementary Figure 13). Interestingly, the expression of Lyn in HS1-positive cell lines was very low, and was much higher in HS1-negative cell lines. These results suggest that HS1 in OCCs may be phosphorylated by kinases other than Lyn. We next tested if tyrosine phosphorylation of HS1 contributed to enhanced cell migration and invasion. Tyrosine residues at 225, 378, and 397 were substituted with phenylalanine and fused to AcGFP to generate AcGFP-HS1**^3YF^** (Figure 5B). Cells that constitutively expressed wild-type AcGFP-HS1 or AcGFP-HS1^3YF^ were established and then siRNA that targeted the 3′-UTR of HS1 mRNA was transfected. As shown in Figure 5C, the siRNA reduced the expression of endogenous HS1, but not exogenously expressed HS1. The migration and invasion of AcGFP-HS1^3YF^ cells were clearly reduced by the depletion of endogenous HS1, whereas AcGFP-HS1 cells did not show a significant reduction in either migration or invasion (Figure 5D). These results indicate that tyrosine phosphorylation of HS1 promotes migration and invasion of OCCs.

## DISCUSSION

We evaluated approximately 60 gene-related factors that have been thought to contribute to EMT in the OCC line ES2. Then, we focused on HS1 due to its homology with CTTN. Many reports have demonstrated the contribution of CTTN to cell migration and invasion in solid tumors [8–10, 13]. HS1 and CTTN have very similar intracellular structures and functions. CTTN is ubiquitously expressed, except in most hematopoietic cells, whereas HS1 is expressed only in hematopoietic cells. It was reported that in normal cells, CTTN was expressed only in platelets [42, 43] and dendritic cells [44]. In cancer, it was shown that CTTN was over-expressed in B-cell lymphocytic leukemia cells, and that it played a role in the Lyn signaling pathway [45]. HS1 was found to be highly expressed in patients with hematopoietic malignancies, and it correlated with poor survival in this population [25, 26, 46]. Recently, we reported that HS1 expression correlated with poor overall survival in patients with ovarian carcinoma [47], and Liao *et al.* showed that HS1 is a prognostic marker in pancreatic ductal adenocarcinoma patients [48]. However, the expression, localization, and role of HS1 in solid tumors have remained unclear.

Our clinicopathological analysis showed that HS1 was over-expressed in ovarian cancer tissues and its expression was closely correlated with poor OS in patients with stage II–IV ovarian cancer, but not in those with stage I disease (Figure 1). From stage II on, OCCs spread beyond the ovaries [49]. We found that HS1 contributed strongly to the invasion and migration abilities of these cells in ovarian cancer patients. Furthermore, we showed that HS1 was expressed in various types of epithelial ovarian cancer (Figure 1A). HS1 may be a very useful biomarker in patients with ovarian cancer.

A review showed that CTTN plays an important role in migration and invasion in both normal and cancer cells [50]. HS1 also contributes to the transendothelial migration of natural killer cells [23]. However, there have been no reports thus far on whether HS1 contributes to cell migration and invasion in non-hematopoietic cells. Here we showed that HS1 contributes strongly to migration and invasion abilities in OCCs (Figures 3 and 5). ES2 and NOE cells express both HS1 and CTTN (Figure 2A and Supplementary Figure 8). The migration and invasion abilities of ES2 and NOE cells decreased after transfection with siRNA against HS1 or CTTN, but the decreases were greater in cells with knocked down HS1 (Figure 3D and Supplementary Figure 10), suggesting that HS1 plays a greater role in migration and invasion abilities than CTTN. HS1 may significantly contribute to migration, invasion, and tumorigenicity *in vivo* but research studies have not yet explored this question. We speculate that CTTN obscures these functions of HS1, which explains why there have been no reports regarding the role of HS1 in solid tumors.

CTTN has three tyrosine residues in its proline-rich region, and phosphorylation of these residues is required for CTTN to influence cell migration and invasion [51, 52]. Similarly, HS1 contains three tyrosine residues whose phosphorylation is necessary to allow HS1 to contribute to the transendothelial migration of natural killer cells [23]. We showed for the first time that HS1 is phosphorylated in OCCs (Supplementary Figure 6A and Supplementary Figure 12), and also that phosphorylation of the tyrosine residues in HS1 is required for its role in cellular invasion and migration abilities. It has been shown that many molecules are involved in invadopodia formation [53]. These include CTTN, cofilin, and TKS5, and their phosphorylation is necessary for this function [50, 54, 55]. Our investigations revealed that HS1, which was thought to be expressed only in hematopoietic lineage cells, is also expressed in OCCs, and also that HS1 plays an important role in OCC migration and invasion. However, in the HS1^3YF^ mutant we developed, all three tyrosine residues were changed to phenylalanine; thus, it is still unclear which tyrosine residue contributes most significantly to the impact of HS1 on OCC migration and invasion. To answer this question, further experiments will be needed. Nonetheless, our study provides useful information for understanding the mechanisms of peritoneal dissemination and metastasis in ovarian carcinomas.

HS1 is also called hematopoietic cell-specific Lyn substrate-1. Lyn is an important kinase in the BCR signaling pathway, and has been reported to be over-expressed and activated in leukemic cells [56]. It phosphorylates HS1 at Y378 and Y397 [57, 58], and HS1 subsequently plays a role in cytoskeleton organization and receptor signaling regulation of normal hematopoietic cells [41, 58–60]. Tyrosine phosphorylation of multiple proteins is essential for invadopodia formation. HS1 is phosphorylated by Lyn, a Src family tyrosine kinase. Invadopodia were first observed in v-Src-transformed cells, and a number of studies have proved that signal pathways activated by Src family kinases are important in invadopodia formation. HS1 may be phosphorylated by tyrosine kinases, including Src family kinases, and functions as a scaffold or actin-nucleating factor for invadopodia formation. In OCCs, Lyn kinase functions as a signaling molecule with the folate receptor [61], and its phosphorylation was found to be markedly reduced after treatment with the multi-kinase inhibitor dasatinib [62]. While Lyn was expressed in all OCCs used in this paper (Supplementary Figure 13), HS1-positive cell lines showed lower expression levels than HS1-negative cell lines. This suggests that HS1 tyrosine residues in OCCs may be phosphorylated by kinases other than Lyn. Taken together with our results, these findings indicate that HS1 may also play a role as a signal regulator in OCCs. HS1 itself, as well as kinases that regulate its phosphorylation, are expected to be therapeutic targets against malignancies.

Altogether, our results indicate that HS1 is a central regulator of cytoskeleton remodeling that significantly influences cell migration, invasion, and tumor formation in ovarian carcinomas. It is suggested that positive HS1 expression may be a useful biomarker for predicting poor prognosis in patients with ovarian carcinomas. Further research examining the mechanisms of HS1 involvement in tumor invasion, metastasis, and malignancy, including studies involving chemotherapeutic agents, will provide new insights that may significantly impact the development of novel therapeutic strategies for primary ovarian carcinomas and their metastasis.

## MATERIALS AND METHODS

### Cell lines

Established human ovarian cancer cell lines A2780, ES2, NOE, NOS1, NOS2, NOS3, NOS4, OVCAR-3, SK-OV-3, and 293T were grown in D-MEM medium (SIGMA, Tokyo, Japan) supplemented with heat-inactivated fetal bovine serum (FBS: Thermo Fisher Scientific, Yokohama, Japan) and 1% penicillin-streptomycin (Nacalai Tesque, Kyoto, Japan). All cells were cultured at 37° C in a 5% CO_2_ humidified incubator. A2780 was purchased from the European Collection of Authenticated Cell Cultures (ECACC, Salisbury, UK). ES2, OVCAR-3, and SK-OV-3 cells were obtained from the American Type Culture Collection (ATCC, Manassas VA, USA). NOE, NOS1, NOS2, NOS3, and NOS4 cells were established at the Department of Obstetrics and Gynecology, Nagoya University Graduate School of Medicine [63].

### Animals

Inbred CAnN.Cg-Foxn1^nu^/CrlCrlj (nude) mice were purchased from Charles River Laboratories International (Yokohama, Japan). The mice were maintained under specific pathogen-free conditions in the Division Of Experimental Animals, Nagoya University.

### Vectors: RNA interference

pAcGFP1-C1-HS1^WT^ and -HS1^3YF^ are mammalian expression vectors encoding AcGFP, a green fluorescent protein from *Aequorea coerulescens,* fused, respectively, with wild-type HS1 (HS1^WT^) and HS1 Y225, Y378, and Y397 mutated to phenylalanine (HS1^3YF^). pSIREN-RetroQ-DsRed-Express-shControl and -shHS1-1904 are small hairpin RNA (shRNA) expression vectors encoding non-target shRNA and shRNA against HS1 (HS1-1904), respectively. Both pAcGFP1-C1 and shControl pSIREN-RetroQ-DsRed-Express were purchased from Clontech (Takara Bio, Kusatsu, Japan). The sequences of small interfering RNA (siRNA) and shRNA are shown in Supplementary Table 1. All siRNAs were synthesized by and purchased from Hokkaido System Science (Sapporo, Japan).

### Immunoblot analysis: immunoprecipitation

Cells were lysed in sample buffer (62.5 mM Tris-HCl [pH 8.0], 2% sodium dodecyl sulfate [SDS], 10% glycerol, 0.002% bromophenol blue, and 100 mM dithiothreitol [DTT]). These samples were separated thorough SDS-PAGE gels, and blotted onto an Immobilon-P transfer membrane (Melk-Millipore, Tokyo, Japan). The sheets incubated with various antibodies, and then treated with secondary antibodies labeled with horseradish peroxidase (GE Healthcare Life Sciences, Tokyo, Japan). The bound antibodies were visualized with the ECL detection reagent (GE) and analyzed by using the Image Quant LAS4000 Mini imaging system (GE). The monoclonal antibodies (mAbs) utilized were anti-HS1 (CST, Tokyo, Japan), anti-phospho-HS1 (Tyr397) (CST), anti-GAPDH (CST), anti-CTTN (BD, Tokyo, Japan), and anti-Lyn (Bio Legend, Tokyo, Japan). Immunoprecipitation was performed using the following steps. Cells were lysed in a lysis buffer (20 mM Tris-HCl [pH 8.0], 150 mM NaCl, 1% Triton X, 10% glycerol, and protease inhibitor cocktail [Nacalai]) on ice. Lysate was immunoprecipitated using anti-HS1 antibody (CST) then incubated with Protein A Agarose beads (BioVision, Milpitas, CA, USA). The agarose beads were boiled. The precipitated proteins in the supernatant were separated from the agarose by centrifugation. The samples were loaded on SDS–PAGE gels then immunoblots were performed as described above. The mAbs utilized were anti-HS1 (BD) and anti-phosphotyrosine (pTyr) antibody PY20 (Melk-Millipore).

### Quantitative real-time PCR (qPCR)

We used the qPCR method to detect mRNAs in human ovarian cancer cell lines and ovarian surface epithelium. Human ovarian surface epithelial cell total RNA was purchased from Cosmo Bio (Tokyo, Japan). Total RNA was extracted from cell lines with the RNeasy Mini kit (Qiagen, Tokyo, Japan), and subjected to qPCR. cDNA was synthesized from 1 μg of total RNA using a High-Capacity cDNA Reverse Transcription kit (Applied Biosystems) with an oligo-dT primer. Fast SYBER Green Master Mix was used for amplification and the samples were run on the StepOne system (Applied Biosystems). The primers were designed to detect spliced mRNA, and their sequences are shown in Supplementary Table 2. All primers were synthesized by and purchased from Hokkaido System Science.

### Transfections and transductions

All siRNA transfections were carried out using Lipofectamine RNAmax (Thermo Fisher Scientific). The vector transfections were carried out using Lipofectamine 3000 (Thermo Fisher Scientific). pSIREN-RetroQ-DsRed-Express-Control and -HS1-1904 were used for gene silencing. To produce viral for delivery, 293T cells were transfected with pSIREN-RetroQ-DsRed-Express (Clontech), VSV and gag-pol. After 3 days, supernatant was collected and filtrated (0.45 μm). Filtrated supernatant with polybrene (final 5 mg/mL) was added to ES2^luc^ cells and incubated.

### Wound-healing assay

The wound-healing assay used a Culture-Insert 2 Well (Ibidi, Nippon Genetics, Tokyo, Japan). Cells were treated with the desired siRNA, shRNA, or expression vectors. Briefly, a cell suspension (7 × 10^5^ cells/mL) was applied to each well (70 μL/well) and then placed in incubator overnight. The Culture-Insert well was removed gently and the dish was filled with cell-free medium. Images of the wounds were obtained using an inverted microscope CKX41 (Olympus, Tokyo, Japan) at 0 (baseline), 6, or 8 hours, and wound healing was quantified using ImageJ software (NIH, Bethesda, MD, USA). The percentage of the original wound area that had healed was defined as follows: [(area of original wound – area at subsequent time point) / area of original wound] × 100 (%).

### Transwell migration and invasion assays

The migration assay was performed in a trans-well chamber (8 mm, Corning Japan, Tokyo, Japan). The invasion assay used a BioCoat Matrigel Invasion Chamber (8 mm, Corning). Cells were treated with the desired siRNA, shRNA, or expression vectors. Briefly, cell suspensions (5 × 10^4^ cells/mL) were placed on top of the upper chamber (500 μL/well). During the 22-hour incubation period, the cells moved through the membrane or matrix, and adhered to the bottom membrane of the insert. Motile cells were fixed with methanol and then stained with May-Grunwald-Giemsa. Images were obtained using an Olympus upright microscope and analyzed using ImageJ software.

### Invadopodia formation assay

NOE cells were transfected with siRNAs. After 48 hours, transfected NOE cells were detached and counted with the ViCell analyzer (Beckman Coulter, Tokyo, Japan). The cells were re-suspended and cultured overnight on gelatin-FITC (Elastin Products Company, Owensville, MO, USA) in the presence of 5-nM metalloproteinase (MMP) inhibitor (GM6001, Cayman Chemical, Ann Arbor, MI, USA). Supernatant was removed, the cells were washed with phosphate buffered saline, and culture medium without MMP inhibitor was added. After 6-hour culture, the cells were fixed and stained with phalloidin-594 (for F-actin; Thermo Fisher Scientific) and DAPI (for nuclei). The cells were then thoroughly washed, placed on glass slides, overlaid with mounting medium (Cosmo Bio), and analyzed using an FV1000 confocal laser scanning microscope (Olympus). In each image, the total number of cells and the number of invadopodia per cell were counted. The invadopodia were identified as gelatin degradation spots (black spots) that co-localized with F-actin (red spots). Invadopodia-positive cells were defined as having over 10 invadopodia. The percentage of invadopodia-positive cells was calculated as follows: [(total number of cells – number of invadopodia-positive cells) / total number of cells] × 100 (%).

### Immunofluorescence

Cells were incubated on glass coverslips. After demonstrating appropriate attachment, the cells were fixed using 4% PFA in PBS. They were then washed with PBS and treated with blocking buffer (5% goat serum and 0.3% Triton-X in PBS) at room temperature for 1 hour and incubated overnight with anti-HS1 antibody in antibody dilution buffer (1% BSA and 0.3% Triton-X in PBS) at 4° C. The coverslips were washed three times with PBS and incubated with Alexa Fluor 488-conjugated secondary antibody (Jackson ImmunoResearch, West Grove, PA, USA), phalloidin-594, and DAPI for 1 hour. After a thorough washing, the cells on coverslips were overlaid with mounting medium (Cosmo Bio), then analyzed with an FV1000 (Olympus).

### Orthotopic inoculation of mice with ovarian cancer cells

ES2^luc^-shControl or -shHS1 cells were inoculated into the ovaries of nude mice. This orthotopic inoculation was described previously [32]. Briefly, female nude mice at 8 weeks of age were used. The cells (1 × 10^5^ cells/μL in medium/Matrigel (1:1) solution) were injected into murine ovaries (1 μL/ovary/mouse) through a retroperitoneal approach from the dorsal flank. Two weeks after inoculation, tumor growth was visualized by intraperitoneal injection with luciferin (1.5 μg/100 mL / 10 g body weight, Promega, Tokyo, Japan) and analyzed with an IVIS Imaging System (PerkinElmer, Waltham, MA, USA).

### Immunohistochemistry

Tissues were obtained from 171 patients who underwent initial surgery at Nagoya University Hospital after providing informed consent. Immunohistochemistry was performed with rabbit mAb against HS1 (CST). The histological type was specified according to the criteria of the World Health Organization classification. The slides were counterstained with Meyer's hematoxylin (Wako, Osaka, Japan). Negative controls were run on all sections in blocking buffer, generated against unrelated antigens. The intensity of immunostaining with HS1 was scored as follows: 0 (negative), 1 (weak), 2 (medium), and 3 (strong). Tumors with a final staining score of 0–1 or 2–3 were defined as having low or high expression of HS1, respectively. The scoring procedure was performed twice by two independent observers; each was blinded to the other's scores and neither had any knowledge of the clinical parameters or other prognostic factors. The interobserver concordance rate was over 95%.

### Statistical analysis

Significant differences between groups were confirmed using the Student's *t*-test. The association of low versus high expression of HS1 and clinicopathological parameters was evaluated using χ^2^-tests. Overall survival was defined as the duration between the date of initial treatment and the last date of follow-up or death from any cause. The correlations between HS1 expression and other pathological variables were assessed using univariate and multivariate Cox hazards analyses. *P* < 0.05 was considered to be statistically significant.

## SUPPLEMENTARY MATERIALS FIGURES AND TABLES



## References

[1] Jiang WG, Sanders AJ, Katoh M, Ungefroren H, Gieseler F, Prince M, Thompson SK, Zollo M, Spano D, Dhawan P, Sliva D, Subbarayan PR, Sarkar M (2015). Tissue invasion and metastasis: Molecular, biological and clinical perspectives. Semin Cancer Biol.

[2] Lambert AW, Pattabiraman DR, Weinberg RA (2017). Emerging Biological Principles of Metastasis. Cell.

[3] Short CA, Suarez-Zayas EA, Gomez TM (2016). Cell adhesion and invasion mechanisms that guide developing axons. Curr Opin Neurobiol.

[4] Pandya P, Orgaz JL, Sanz-Moreno V (2017). Modes of invasion during tumour dissemination. Mol Oncol.

[5] Te Boekhorst V, Friedl P (2016). Plasticity of Cancer Cell Invasion-Mechanisms and Implications for Therapy. Adv Cancer Res.

[6] Gomathinayagam R, Muralidharan J, Ha JH, Varadarajalu L, Dhanasekaran DN (2014). Hax-1 is required for Rac1-Cortactin interaction and ovarian carcinoma cell migration. Genes Cancer.

[7] Zhang Y, Zhang M, Dong H, Yong S, Li X, Olashaw N, Kruk PA, Cheng JQ, Bai W, Chen J, Nicosia SV, Zhang X (2009). Deacetylation of cortactin by SIRT1 promotes cell migration. Oncogene.

[8] MacGrath SM, Koleske AJ (2012). Cortactin in cell migration and cancer at a glance. J Cell Sci.

[9] Weaver AM (2008). Cortactin in tumor invasiveness. Cancer Lett.

[10] Siar CH, Rahman ZA, Tsujigiwa H, Mohamed Om Alblazi K, Nagatsuka H, Ng KH (2016). Invadopodia proteins, cortactin, N-WASP and WIP differentially promote local invasiveness in ameloblastoma. J Oral Pathol Med.

[11] Clark ES, Whigham AS, Yarbrough WG, Weaver AM (2007). Cortactin is an essential regulator of matrix metalloproteinase secretion and extracellular matrix degradation in invadopodia. Cancer Res.

[12] Bryce NS, Clark ES, Leysath JL, Currie JD, Webb DJ, Weaver AM (2005). Cortactin promotes cell motility by enhancing lamellipodial persistence. Curr Biol.

[13] Wu H, Cheng X, Ji X, He Y, Jing X, Wu H, Zhao R (2016). Cortactin contributes to the tumorigenicity of colorectal cancer by promoting cell proliferation. Oncol Rep.

[14] Ni QF, Yu JW, Qian F, Sun NZ, Xiao JJ, Zhu JW (2015). Cortactin promotes colon cancer progression by regulating ERK pathway. Int J Oncol.

[15] Hou H, Chen W, Zhao L, Zuo Q, Zhang G, Zhang X, Wang H, Gong H, Li X, Wang M, Wang Y, Li X (2012). Cortactin is associated with tumour progression and poor prognosis in prostate cancer and SIRT2 other than HADC6 may work as facilitator *in situ*. J Clin Pathol.

[16] Takemoto Y, Furuta M, Li XK, Strong-Sparks WJ, Hashimoto Y (1995). LckBP1, a proline-rich protein expressed in haematopoietic lineage cells, directly associates with the SH3 domain of protein tyrosine kinase p56lck. EMBO J.

[17] Kitamura D, Kaneko H, Miyagoe Y, Ariyasu T, Watanabe T (1989). Isolation and characterization of a novel human gene expressed specifically in the cells of hematopoietic lineage. Nucleic Acids Res.

[18] Schuuring E, van Damme H, Schuuring-Scholtes E, Verhoeven E, Michalides R, Geelen E, de Boer C, Brok H, van Buuren V, Kluin P (1998). Characterization of the EMS1 gene and its product, human Cortactin. Cell Adhes Commun.

[19] Fischer U, Michel A, Meese EU (2005). Expression of the gene for hematopoietic cell specific protein is not restricted to cells of hematopoietic origin. Int J Mol Med.

[20] Yamanashi Y, Fukuda T, Nishizumi H, Inazu T, Higashi K, Kitamura D, Ishida T, Yamamura H, Watanabe T, Yamamoto T (1997). Role of tyrosine phosphorylation of HS1 in B cell antigen receptor-mediated apoptosis. J Exp Med.

[21] Gomez TS, McCarney SD, Carrizosa E, Labno CM, Comiskey EO, Nolz JC, Zhu P, Freedman BD, Clark MR, Rawlings DJ, Billadeau DD, Burkhardt JK (2006). HS1 functions as an essential actin-regulatory adaptor protein at the immune synapse. Immunity.

[22] Uruno T, Zhang P, Liu J, Hao JJ, Zhan X (2003). Haematopoietic lineage cell-specific protein 1 (HS1) promotes actin-related protein (Arp) 2/3 complex-mediated actin polymerization. Biochem J.

[23] Mukherjee S, Kim J, Mooren OL, Shahan ST, Cohan M, Cooper JA (2015). Role of cortactin homolog HS1 in transendothelial migration of natural killer cells. PLoS One.

[24] Scielzo C, Bertilaccio MT, Simonetti G, Dagklis A, ten Hacken E, Fazi C, Muzio M, Caiolfa V, Kitamura D, Restuccia U, Bachi A, Rocchi M, Ponzoni M (2010). HS1 has a central role in the trafficking and homing of leukemic B cells. Blood.

[25] Butrym A, Majewski M, Dzietczenia J, Kuliczkowski K, Mazur G (2012). High expression of hematopoietic cell specific Lyn substrate-1 (HS1) predicts poor survival of B-cell chronic lymphocytic leukemia patients. Leuk Res.

[26] Frezzato F, Gattazzo C, Martini V, Trimarco V, Teramo A, Carraro S, Cabrelle A, Ave E, Facco M, Zambello R, Tibaldi E, Brunati AM, Semenzato G, Trentin L (2012). HS1, a Lyn kinase substrate, is abnormally expressed in B-chronic lymphocytic leukemia and correlates with response to fludarabine-based regimen. PLoS One.

[27] Torre LA, Bray F, Siegel RL, Ferlay J, Lortet-Tieulent J, Jemal A (2015). Global cancer statistics, 2012. CA Cancer J Clin.

[28] Yamagami W, Nagase S, Takahashi F, Ino K, Hachisuga T, Aoki D, Katabuchi H (2017). Clinical statistics of gynecologic cancers in Japan. J Gynecol Oncol.

[29] Lengyel E (2010). Ovarian cancer development and metastasis. Am J Pathol.

[30] Brooks SA, Lomax-Browne HJ, Carter TM, Kinch CE, Hall DM (2010). Molecular interactions in cancer cell metastasis. Acta Histochem.

[31] Coffman LG, Burgos-Ojeda D, Wu R, Cho K, Bai S, Buckanovich RJ (2016). New models of hematogenous ovarian cancer metastasis demonstrate preferential spread to the ovary and a requirement for the ovary for abdominal dissemination. Transl Res.

[32] Koya Y, Kajiyama H, Liu W, Shibata K, Senga T, Kikkawa F (2016). Murine Experimental Model of Original Tumor Development and Peritoneal Metastasis via Orthotopic Inoculation with Ovarian Carcinoma Cells. J Vis Exp.

[33] Zhang Y, Fan N, Yang J (2015). Expression and clinical significance of hypoxia-inducible factor 1alpha, Snail and E-cadherin in human ovarian cancer cell lines. Mol Med Rep.

[34] Jeannot P, Nowosad A, Perchey RT, Callot C, Bennana E, Katsube T, Mayeux P, Guillonneau F, Manenti S, Besson A (2017). p27Kip1 promotes invadopodia turnover and invasion through the regulation of the PAK1/Cortactin pathway. eLife.

[35] Jing X, Wu H, Ji X, Wu H, Shi M, Zhao R (2016). Cortactin promotes cell migration and invasion through upregulation of the dedicator of cytokinesis 1 expression in human colorectal cancer. Oncol Rep.

[36] Li Y, Tondravi M, Liu J, Smith E, Haudenschild CC, Kaczmarek M, Zhan X (2001). Cortactin potentiates bone metastasis of breast cancer cells. Cancer Res.

[37] Liang J, Li X, Li Y, Wei J, Daniels G, Zhong X, Wang J, Sfanos K, Melamed J, Zhao J, Lee P (2015). LEF1 targeting EMT in prostate cancer invasion is mediated by miR-181a. Am J Cancer Res.

[38] Santiago L, Daniels G, Wang D, Deng FM, Lee P (2017). Wnt signaling pathway protein LEF1 in cancer, as a biomarker for prognosis and a target for treatment. Am J Cancer Res.

[39] Wu DI, Liu L, Ren C, Kong D, Zhang P, Jin X, Wang T, Zhang G (2016). Epithelial-mesenchymal interconversions and the regulatory function of the ZEB family during the development and progression of ovarian cancer. Oncol Lett.

[40] Zhang P, Sun Y, Ma L (2015). ZEB1: at the crossroads of epithelial-mesenchymal transition, metastasis and therapy resistance. Cell Cycle.

[41] Butler B, Kastendieck DH, Cooper JA (2008). Differently phosphorylated forms of the cortactin homolog HS1 mediate distinct functions in natural killer cells. Nat Immunol.

[42] Thomas SG, Poulter NS, Bem D, Finney B, Machesky LM, Watson SP (2017). The actin binding proteins cortactin and HS1 are dispensable for platelet actin nodule and megakaryocyte podosome formation. Platelets.

[43] Zhan X, Haudenschild CC, Ni Y, Smith E, Huang C (1997). Upregulation of cortactin expression during the maturation of megakaryocytes. Blood.

[44] Van Audenhove I, Debeuf N, Boucherie C, Gettemans J (2015). Fascin actin bundling controls podosome turnover and disassembly while cortactin is involved in podosome assembly by its SH3 domain in THP-1 macrophages and dendritic cells. Biochim Biophys Acta.

[45] Gattazzo C, Martini V, Frezzato F, Trimarco V, Tibaldi E, Castelli M, Facco M, Zonta F, Brunati AM, Zambello R, Semenzato G, Trentin L (2014). Cortactin, another player in the Lyn signaling pathway, is over-expressed and alternatively spliced in leukemic cells from patients with B-cell chronic lymphocytic leukemia. Haematologica.

[46] Scielzo C, Ghia P, Conti A, Bachi A, Guida G, Geuna M, Alessio M, Caligaris-Cappio F (2005). HS1 protein is differentially expressed in chronic lymphocytic leukemia patient subsets with good or poor prognoses. J Clin Invest.

[47] Liu W, Kajiyama H, Shibata K, Koya Y, Senga T, Kikkawa F (2018). Hematopoietic lineage cell-specific protein 1 immunoreactivity indicates an increased risk of poor overall survival in patients with ovarian carcinoma. Oncol Lett.

[48] Liao X, Huang K, Huang R, Liu X, Han C, Yu L, Yu T, Yang C, Wang X, Peng T (2017). Genome-scale analysis to identify prognostic markers in patients with early-stage pancreatic ductal adenocarcinoma after pancreaticoduodenectomy. OncoTargets Ther.

[49] Heintz AP, Odicino F, Maisonneuve P, Beller U, Benedet JL, Creasman WT, Ngan HY, Sideri M, Pecorelli S (2001). Carcinoma of the ovary. J Epidemiol Biostat.

[50] Kirkbride KC, Sung BH, Sinha S, Weaver AM (2011). Cortactin: a multifunctional regulator of cellular invasiveness. Cell Adhes Migr.

[51] Magalhaes MA, Larson DR, Mader CC, Bravo-Cordero JJ, Gil-Henn H, Oser M, Chen X, Koleske AJ, Condeelis J (2011). Cortactin phosphorylation regulates cell invasion through a pH-dependent pathway. J Cell Biol.

[52] Lee MS, Kim S, Kim BG, Won C, Nam SH, Kang S, Kim HJ, Kang M, Ryu J, Song HE, Lee D, Ye SK, Jeon NL (2014). Snail1 induced in breast cancer cells in 3D collagen I gel environment suppresses cortactin and impairs effective invadopodia formation. Biochim Biophys Acta.

[53] Murphy DA, Courtneidge SA (2011). The ‘ins’ and ‘outs’ of podosomes and invadopodia: characteristics, formation and function. Nat Rev Mol Cell Biol.

[54] Nagai S, Moreno O, Smith CA, Ivanchuk S, Romagnuolo R, Golbourn B, Weeks A, Seol HJ, Rutka JT (2011). Role of the cofilin activity cycle in astrocytoma migration and invasion. Genes Cancer.

[55] Burger KL, Learman BS, Boucherle AK, Sirintrapun SJ, Isom S, Diaz B, Courtneidge SA, Seals DF (2014). Src-dependent Tks5 phosphorylation regulates invadopodia-associated invasion in prostate cancer cells. Prostate.

[56] Contri A, Brunati AM, Trentin L, Cabrelle A, Miorin M, Cesaro L, Pinna LA, Zambello R, Semenzato G, Donella-Deana A (2005). Chronic lymphocytic leukemia B cells contain anomalous Lyn tyrosine kinase, a putative contribution to defective apoptosis. J Clin Invest.

[57] Trentin L, Frasson M, Donella-Deana A, Frezzato F, Pagano MA, Tibaldi E, Gattazzo C, Zambello R, Semenzato G, Brunati AM (2008). Geldanamycin-induced Lyn dissociation from aberrant Hsp90-stabilized cytosolic complex is an early event in apoptotic mechanisms in B-chronic lymphocytic leukemia. Blood.

[58] Brunati AM, Donella-Deana A, James P, Quadroni M, Contri A, Marin O, Pinna LA (1999). Molecular features underlying the sequential phosphorylation of HS1 protein and its association with c-Fgr protein-tyrosine kinase. J Biol Chem.

[59] Carrizosa E, Gomez TS, Labno CM, Klos Dehring DA, Liu X, Freedman BD, Billadeau DD, Burkhardt JK (2009). Hematopoietic lineage cell-specific protein 1 is recruited to the immunological synapse by IL-2-inducible T cell kinase and regulates phospholipase Cgamma1 Microcluster dynamics during T cell spreading. J Immunol.

[60] Yamanashi Y, Okada M, Semba T, Yamori T, Umemori H, Tsunasawa S, Toyoshima K, Kitamura D, Watanabe T, Yamamoto T (1993). Identification of HS1 protein as a major substrate of protein-tyrosine kinase(s) upon B-cell antigen receptor-mediated signaling. Proc Natl Acad Sci U S A.

[61] Miotti S, Bagnoli M, Tomassetti A, Colnaghi MI, Canevari S (2000). Interaction of folate receptor with signaling molecules lyn and G(alpha)(i-3) in detergent-resistant complexes from the ovary carcinoma cell line IGROV1. J Cell Sci.

[62] Konecny GE, Glas R, Dering J, Manivong K, Qi J, Finn RS, Yang GR, Hong KL, Ginther C, Winterhoff B, Gao G, Brugge J, Slamon DJ (2009). Activity of the multikinase inhibitor dasatinib against ovarian cancer cells. Br J Cancer.

[63] Umezu T, Kajiyama H, Terauchi M, Shibata K, Ino K, Nawa A, Kikkawa F (2007). Establishment of a new cell line of endometrioid carcinoma of the ovary and its chemosensitivity. Hum Cell.

[64] Zhao WD, Liu W, Fang WG, Kim KS, Chen YH (2010). Vascular endothelial growth factor receptor 1 contributes to Escherichia coli K1 invasion of human brain microvascular endothelial cells through the phosphatidylinositol 3-kinase/Akt signaling pathway. Infect Immun.

