# Supplementary material for: Hematopoietic lineage cell-specific protein 1 (HS1), a hidden player in migration, invasion, and tumor formation, is over-expressed in ovarian carcinoma cells
Source: Oncotarget. 2018 Aug 24;9(66):32609–23. doi: 10.18632/oncotarget.25975 (PMC6135686; doi:10.18632/oncotarget.25975)
Supplement: Supplementary file 1 [file oncotarget-09-32609-s001.pdf]

Hematopoietic lineage cell-specific protein 1 (HS1), a hidden player in migration, invasion, and tumor formation, is over-expressed in ovarian carcinoma cells

SUPPLEMENTARY MATERIALS

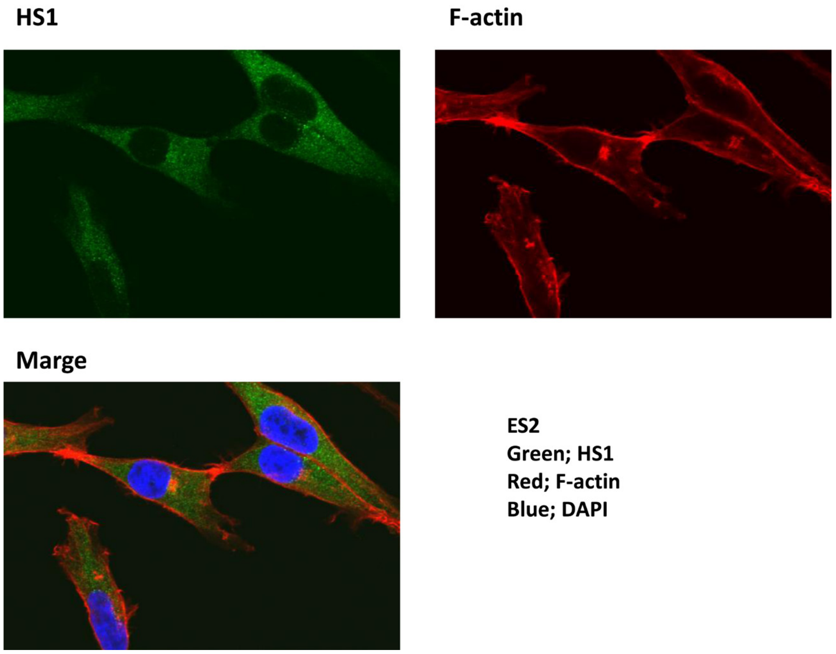

Supplementary Figure 1: HS1 localized in cytoplasm in ovarian cancer cells. Immunostaining for HS1 (green) was detected in cytoplasm and the cells were counterstained with F-actin (red) and DAPI (blue). Photographs were acquired by confocal microscopy.

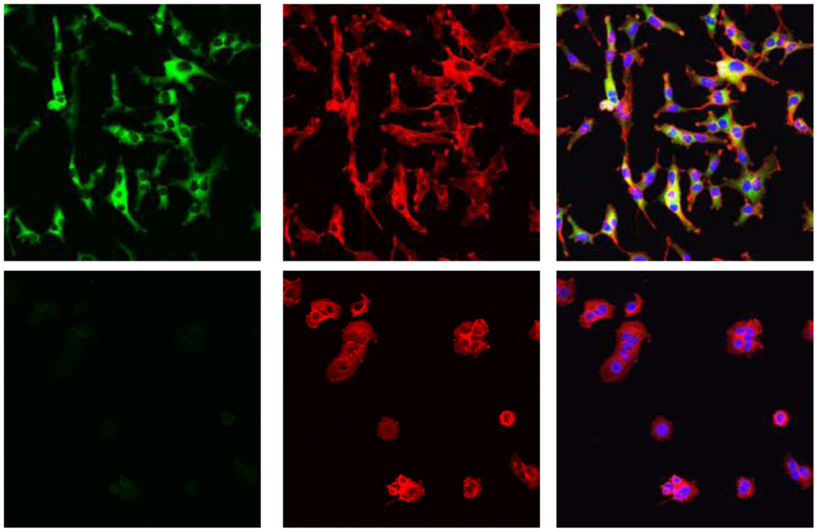

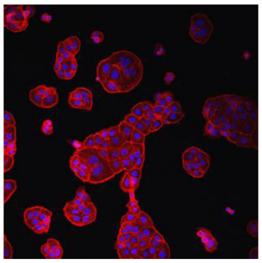

Supplementary Figure 2: Ovarian cancer cell line ES2 showed morphological changes after HS1 knock-down by siRNA. Double immunofluorescence showed that HS1 expression in ES2 cells that were transfected with HS1 siRNA was significantly decreased compared with cells transfected with control siRNA. In ES2 cells transfected with control siRNA, immunostaining for HS1 (green) was detected in cytoplasm and the cells were counterstained with F-actin (red) and DAPI (blue). Ovarian cancer cell line NOS2 was used as an HS1-negative control. Photographs were acquired by confocal microscopy.

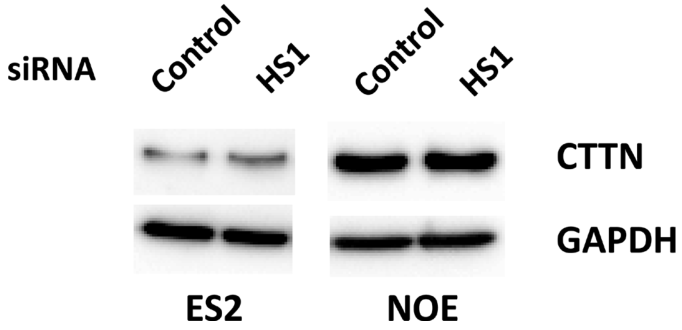

Supplementary Figure 3: HS1 siRNA did not decrease the expression of CTTN. After transfection with HS1 siRNA (HS1-268 and -965, 10 nM each) or control (20 nM), cell lysates were used in immunoblot analysis to confirm whether treatment with HS1 siRNA could decrease CTTN expression.

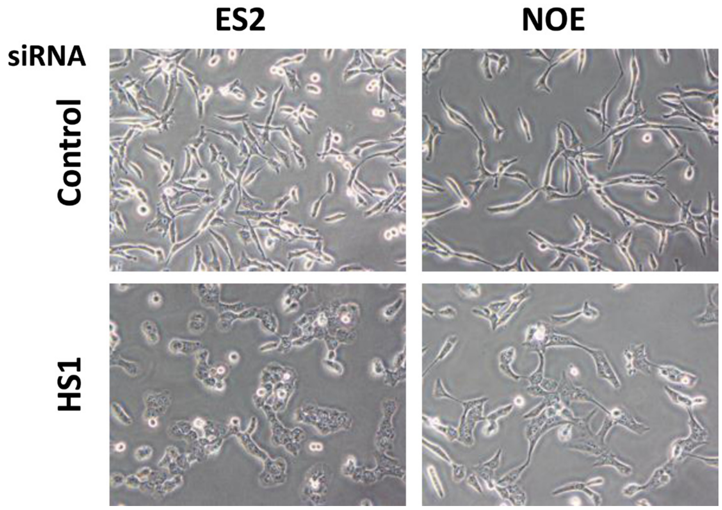

Supplementary Figure 4: Ovarian cancer cell lines ES2 and NOE showed morphological changes after transfection with HS1 siRNA but not control siRNA.

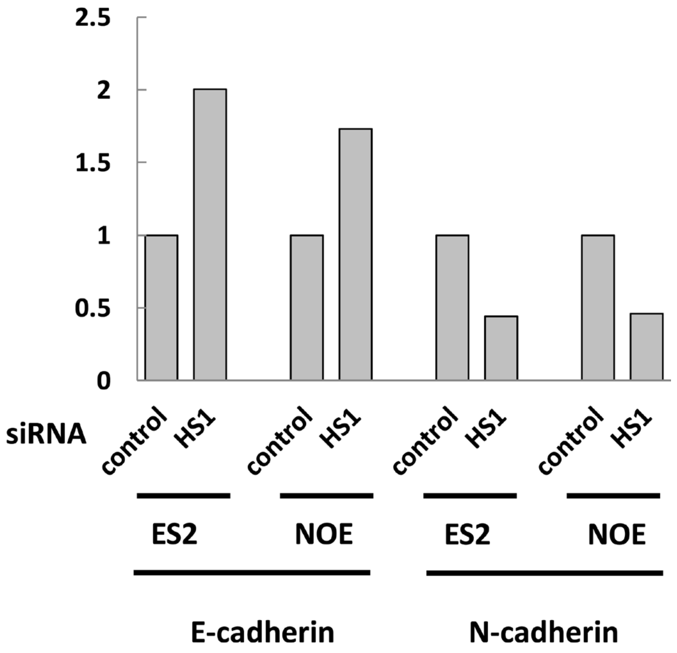

Supplementary Figure 5: The expression of E- and N-cadherin mRNAs was altered in ES2 and NOE cells after HS1 knock-down. After transfection of ES2 and NOE cells with HS1 siRNA (HS1-268 and -965, 10 nM each) or control (20 nM), total RNA was isolated from cells and used to synthesize cDNA. Quantitative real-time PCR was used to measure the expression levels of the *E-* and *N-cadherin* genes (cells transfected with control siRNA were used as a reference, with a value of 1). *GAPDH* was used for normalization.

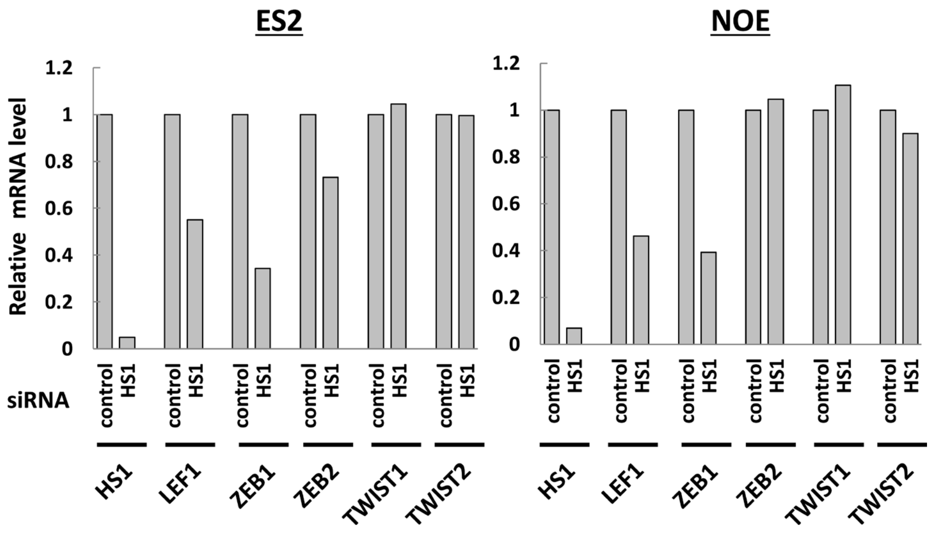

Supplementary Figure 6: The expression of LEF1 and ZEB1 mRNAs was reduced in ES2 and NOE cells after transfection with HS1siRNA. After transfection of ES2 and NOE cells with HS1 siRNA (HS1-268 and -965, 10 nM each) or control (20 nM), total RNA was isolated from the cells and used to synthesize cDNA. Quantitative real-time PCR was used to measure expression levels of the *HS1*, *LEF1*, *ZEB1*, *ZEB2*, *TWIST1*, and *TWIST2* genes (cells transfected with control siRNA were used as a reference, with a value of 1). *GAPDH* was used for normalization.

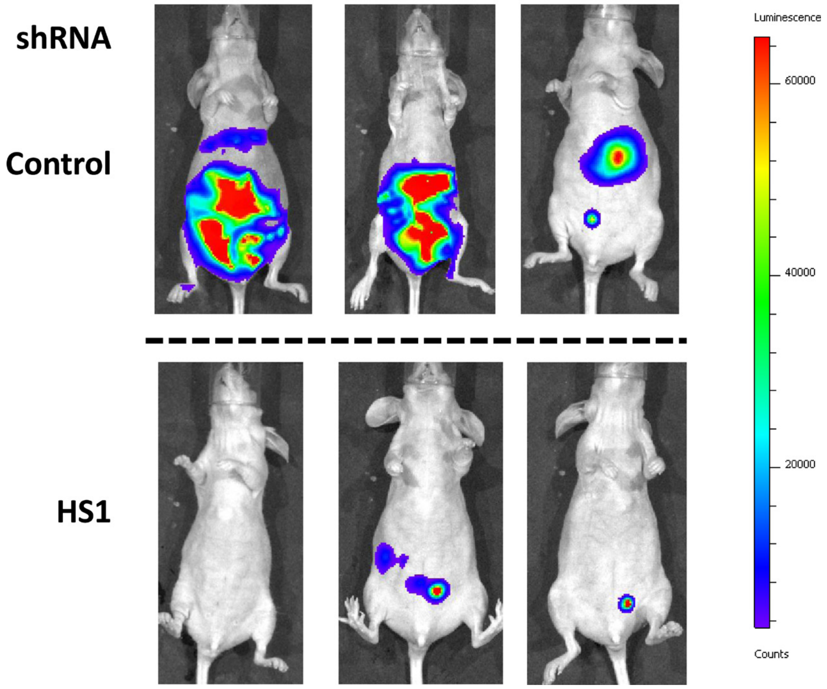

Supplementary Figure 7: Intraperitoneal inoculation was used as a model of peritoneal dissemination. Briefly, 5-week-old female nude mice were inoculated intraperitoneally with ES2-shControl or ES2-shHS1 cells (5×10^5 cells/100 µL/mouse). Two weeks after inoculation, tumor growth was visualized by intraperitoneal injection with luciferin (1.5 mg/100 µL/10 g body weight) and analyzed with an IVIS Imaging System. The upper row shows mice inoculated with ES2-shControl and the lower row shows mice inoculated with ES2-shHS1.

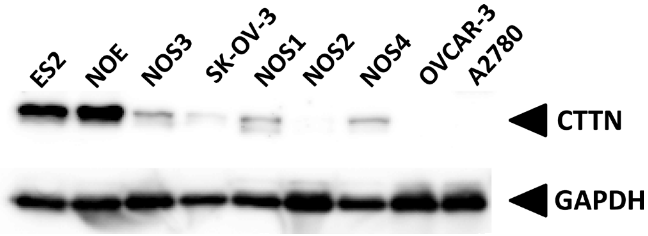

Supplementary Figure 8: CTTN is expressed in cells with highly invasive features. Cells were lysed and used to perform immunoblot analysis with an antibody against CTTN. GAPDH was used as a loading control.

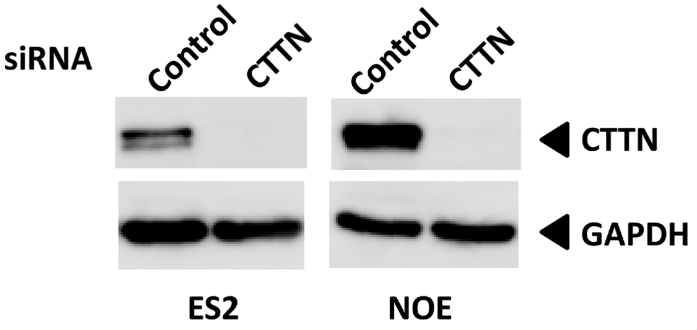

Supplementary Figure 9: Expression of CTTN was efficiently suppressed after transfection with CTTN siRNA in both ES2 and NOE cells. After transfection with CTTN siRNA (CTTN-377 and -508, 10 nM each) or control (20 nM), cell lysates were used to perform immunoblot analysis to confirm knock-down of CTTN. These CTTN siRNAs did not decrease the expression of HS1 (data not shown).

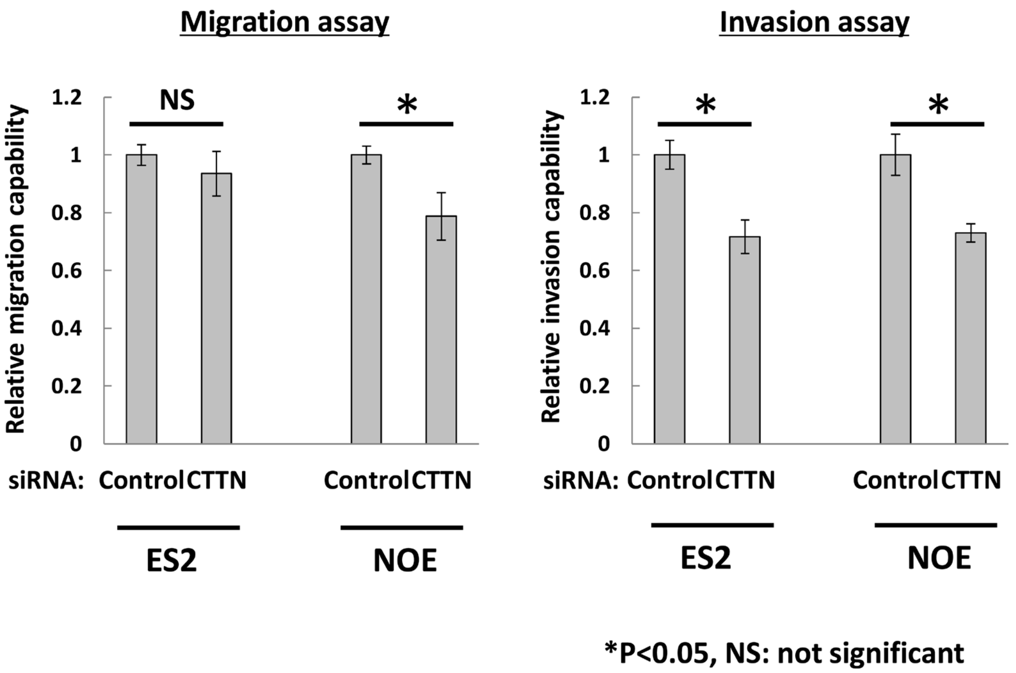

Supplementary Figure 10: Transwell migration and invasion assays after transfection with CTTN siRNA. ES2 and NOE cells were transfected with CTTN siRNA or control, and then transwell migration and invasion assays were performed. After 22 hours, cells were fixed and stained and then photographs were acquired. The migrating and invading cells were counted using ImageJ software, and the relative migration or invasive abilities are shown (cells transfected with control siRNA were used as a reference, with a value of 1). Each experiment was performed in triplicate. The bars indicate the mean ± s.d. $^{*}P < 0.05$, NS: not significant (*t*-test).

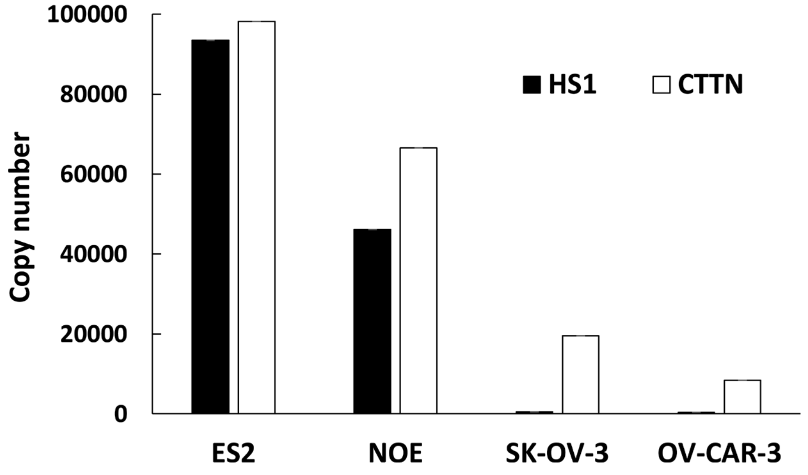

Supplementary Figure 11: The levels of HS1 and CTTN mRNAs in ES2 and NOE cells. To compare the gene expression of *HS1* with that of *CTTN*, absolute qPCR quantification was performed to determine the absolute copy number of these genes in ES2, NOE, SK-OV-3, and OV-CAR-3 cells using the StepOne system (Applied Biosystems). The standard curves were constructed from a diluted standard template using pGEM-T-GAPDH. This GAPDH fragment was amplified using GAPDH primers (Supplementary Table 2) then cloned into the pGEM-T vector (Promega).

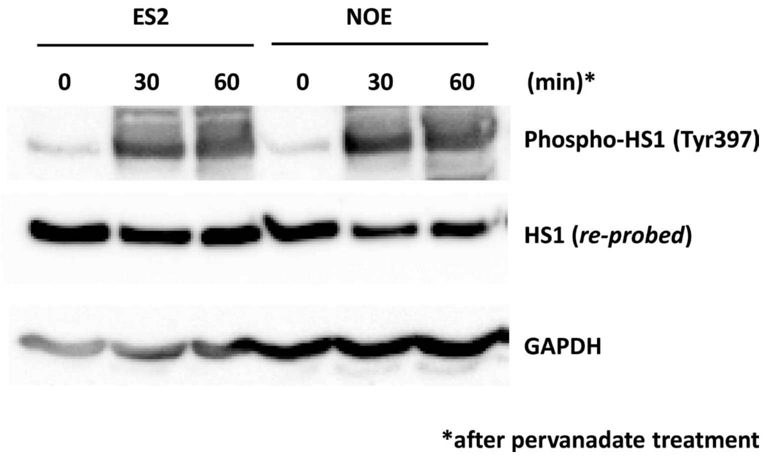

Supplementary Figure 12: Phosphorylation of the tyrosine residue at position 397 (Tyr397) in HS1. After pervanadate treatment (final concentration 100 mM), lysates extracted from treated ES2 and NOE cells were used to perform immunoblot analysis with an antibody against phosphor-HS1 (Tyr397). After stripping treatment, the membrane was re-used for immunoblot analysis with antibody against HS1. GAPDH was used as a loading control.

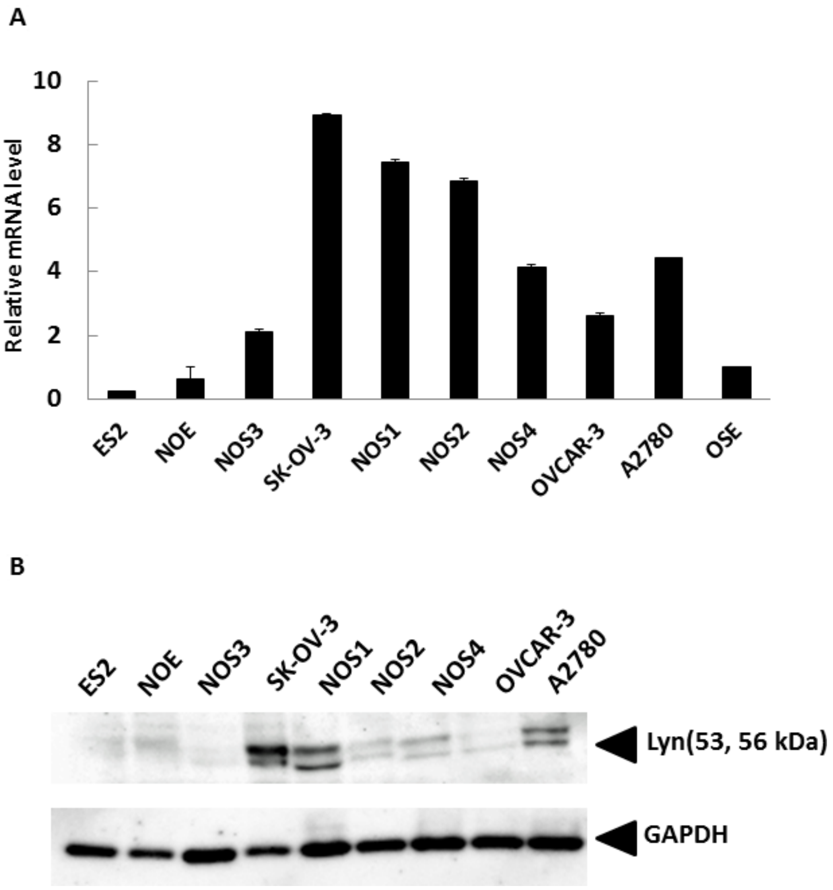

Supplementary Figure 13: Expression of Lyn in OCCs. (**A**) Total RNA was isolated from cells and used to synthesize cDNA. Quantitative PCR was used to measure the expression levels of the *Lyn* gene (OSE was used as a reference, with a value of 1). *GAPDH* was used for normalization. (**B**) Cells were lysed and immunoblot analysis was performed with an antibody against Lyn. GAPDH was used as a loading control.

Supplementary Table1: The sequences of siRNA and shRNA used in this study

Target gene	siRNA	Target sequence (5′ to 3′)
non-targeted	control	TTCTCCGAACGTGTCACGT
HS1	HS1-268	GCACAGAACACATCAACAT
	HS1-965	GCCAGTGATAGCTATGGAA
	HS1-1788	GCTCATGAACATTTCTCTT
CTTN	CTNN-377	GCTGAGGGAGAATGTCTTT
	CTNN-508	GCCACGAATATCAGTCGAA

Target gene	siRNA	Target sequence (5′ to 3′)
non-targeted	control	TTCTCCGAACGTGTCACGT
HS1	HS1-1904	GGGAAAGTACGTCTAGATTGTGT

Supplementary Table 2: The sequences of qPCR primers used in this study

Target gene	siRNA	Target sequence (5′ to 3′)
HS1	HS1-Fw	CCTTGCTGCCCATTAGGCAGAC
	HS1-Rev	CAGAGTCCTAGGGGGCAGAGC
GAPDH	GAPDH-Fw	CATGTTCGTCATGGGTGTGAACCA
	GAPDH-Rev	AGTGATGGCATGGACTGTGGTCAT
E-cadherin	Ecad-Fw	TGAGTGTCCCCCGGTATCTTC
	Ecad-Rev	CAGTATCAGCCGCTTTCAGATTT
N-cadherin	Ncad-Fw	TGCTGTTTTGGACCGAGAATCACC
	Ncad-Rev	CAGCGTTCCTGTTCCACTCATAGG
LEF1	LEF1-Fw	CCCCGATGACGGAAAGCATC
	LEF1-Rev	TGTTCTCGGGATGGGTGGAGAA
TWIST1	TWIST1-Fw	CACCCAGTCGCTGAACGAGGC
	TWIST1-Rev	CTGCAGCTTGCCATCTTGGAGTC
TWIST2	TWIST2-Fw	AGCCCCAGCGCGCAGTCCTT
	TWIST2-Rev	AGCGCCGCGAAGGCCTCGTT
ZEB1	ZEB1-Fw	GCACAACCAAGTGCAGAAGA
	ZEB1-Rev	CATTTGCAGATTGAGGCTA
ZEB2	ZEB2-Fw	CCAGCGGAAACAAGGATTTCAG
	ZEB2-Rev	ACAGGAGTCGGAGTCTGTCA
CTTN	CTTN-Fw	CCGGGCACCAGGAGCATATC
	CTNN-Rev	CGTGGCCGACAGCTGACTTATC
